# Melatonin: a positive influencer of inflammation in neonatal encephalopathy

**DOI:** 10.1038/s41390-025-03876-7

**Published:** 2025-01-20

**Authors:** Raymand Pang, Eleanor Molloy, Nicola J. Robertson

**Affiliations:** 1 Institute for Women’s Health, University College London, WC1E 6HX, 74 Huntley Street, London, WC1E 6HX UK; 2 Discipline of Paediatrics, Trinity College Dublin, The University of Dublin, Trinity Translational Medicine Institute (TTMI), St James Hospital & Trinity Research in Childhood Centre (TRiCC), Dublin, Ireland; 3 Neonatology & Neurodisability, Children’s Hospital Ireland (CHI), Dublin, Ireland; 4 Paediatrics, Coombe Women’s and Infant’s University Hospital, Dublin, Ireland; 5 Centre for Clinical Brain Sciences, University of Edinburgh, Edinburgh, UK

Momentum continues in the search for effective and safe neuroprotective therapies for neonatal encephalopathy (NE), despite recent disappointing results of the HEAL,^1^ PAEAN (https://ctc.usyd.edu.au/our-research/research-areas/neonatal-and-perinatal/in-follow-up/paean-study/) and Albino Trials.^2^ Calero et al.^3^ report an exciting but preliminary study suggesting that adding intravenous melatonin to therapeutic hypothermia (HT) in newborns with NE reduces plasma pro-inflammatory levels, leading to improved long-term neurodevelopmental outcomes. This was a small trial, involving 25 newborns with NE meeting criteria for HT. Babies were randomly assigned to receive either HT plus placebo (0.9% sodium chloride) or HT plus intravenous (IV) melatonin (5 mg/kg/day) for 3 days. Blood samples were collected at four time points (on hospital admission, 24, 48 and 72 h) to measure the levels of inflammatory biomarkers, including GFAP, GM-CSF, IL-1, IL-2, IL-7 and IL-13. Infants underwent neurodevelopmental assessments at 6 and 18 months of age using the Bayley Scales of Infant and Toddler Development-III, the Gross Motor Function Classification System (GMFCS), and the Tardieu scale. The group have previously reported that IV melatonin appeared safe with no adverse effect on metabolic, renal, hepatic and hematological blood parameters.^4^ In this sub-study, the melatonin group exhibited significantly lower plasma levels of GM-CSF, IL-2, and IL-13 within the first 24 h after birth compared to the placebo group. These lower cytokine levels correlated with better 2 year neurodevelopmental outcome.

The study team acknowledge the limitations of the small sample size, lack of power calculation and potential bias due to a higher severity of NE in the placebo group. In addition, we note that no plasma melatonin levels are reported and it is unclear when the first dose of melatonin was administered. This information is important as preclinical data suggests that melatonin is most effective as a neuroprotective therapy after hypoxia ischemia (HI) when therapeutic levels of 15–30 mg/L are achieved within 8 h after the HI insult.^5,6^ While not explicitly stated in this article, the group previously reported the IV melatonin formulation contains propylene glycol and macrogol as excipients.^4^ In our experience, the use of ethanol as an excipient may be more protective than other excipients.^5–7^ Notwithstanding these points, these data are very encouraging especially as melatonin is currently being studied in an international phase I first in human dose escalation safety study (The ACUMEN Study—*Acute High Dose Melatonin for Encephalopathy of the Newborn)*. In this study the aim is to identify the pharmacokinetic (PK) profile of IV melatonin using a step-wise dose escalation protocol; monitoring comprehensively for adverse effects in ∼60 babies with moderate-severe NE. The outcome is the identification of a recommended phase 2 dose, based on safety and plasma melatonin levels.

A key advantage of melatonin for NE is its positive safety profile and diverse mechanisms of action including anti-oxidative, anti-inflammatory, anti-apoptotic and receptor-mediated effects.^5^ Strong preclinical data support its additional benefit with HT^6–8^ as well as its efficacy as a monotherapy in inflammation-augmented HI.^9^ In NE, following the period of HI, cellular energy production is disrupted because of impaired membrane transport, reduced ATP production, and systemic acidosis. Membrane depolarization facilitates calcium entry and activates N-methyl-D-aspartate receptors, as well as other excitotoxic neurotransmitters. These processes trigger inflammatory cascades, which generate local and systemic cytokine release that recruit white blood cells to the site of central nervous system injury.^10^ Secondary injury is influenced by the severity of this immune response, predisposing to cytotoxic edema, excitotoxicity, activation of apoptotic pathways, and ultimately neuronal death. Proinflammatory cytokines,^11,12^ excitotoxins, and reactive oxygen species (ROS)^13^ are considered key contributors to brain damage resulting from neuroinflammatory processes. Early disruption of this cascade by melatonin is likely to be the critical event in ameliorating the downstream inflammatory cascade, subsequent cell death and brain injury.

**Fig. 1:**
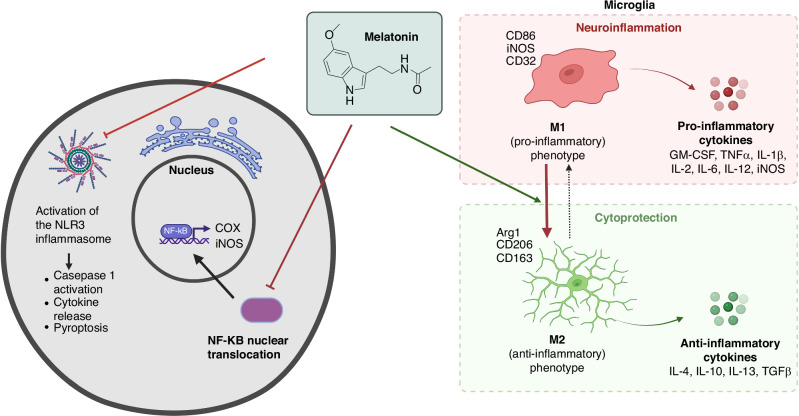
Anti-inflammatory effects of melatonin. Melatonin possesses anti-inflammatory properties by inhibiting NLRP3 inflammasome activation, thus inhibiting caspase-1 activation, cytokine release and pyroptosis. In addition, melatonin inhibits the expression of the cyclooxygenase (COX) and inducible nitric oxide synthase (iNOS) by inhibiting nuclear NF-κB translocation. Melatonin triggers polarization to the anti-inflammatory (M2) phenotype, as shown by the upregulation of M2-specific markers, such as Arg1 and CD206, and disfavors the pro-inflammatory (M1) phenotype, as indicated by the downregulation of iNOS.

Melatonin is a ubiquitous molecule which carries out many essential healing functions in the cell. Melatonin has remarkable versatility, diversity and connection with the immune system, strongly stimulating immune cells,^14^ indicating its potential use as a therapeutic agent.^15,16^ The interactions with the immune system are complex, as both the circadian rhythm (which is stimulated by melatonin) and melatonin itself affect the immune system.^17^ In addition, the immunological actions of melatonin are not uniform, but are conditional and can comprise both pro- and anti-inflammatory effects.^18^ This is not surprising given the many participating cell types, humoral factors, flexible regulatory network, participation of non-immune cells and the feedback loops that occur over time.^19^ However, many studies describe the suppression of proinflammatory signals by melatonin following HI; these include the downregulation of iNOS (inducible nitric oxide and COX-2 (cyclooxygenase-2). Of central importance in the inflammatory cascade is NF-κB signaling pathway, which is essential for the regulation of immunity and inflammation. Excessive inflammatory response after HI is mediated by the activation of the NF-κB pathway and activated NF-kB upregulates the production of proinflammatory cytokines such as IL-1, IL-6, TNF-alpha. Melatonin performs part of its anti-inflammatory functions by modulating nuclear NF-κB translocation.^20^ Furthermore, melatonin is reported to inhibit the activation of the inflammasome,^21^ an innate immune system receptor and sensor that regulates the activation of caspase-1 and induces inflammation in response to HI and other triggers. Indeed, melatonin was observed to reduce LPS-induced inflammation and thus NLRP3 inflammasome formation in mouse adipose tissue by acting on the expression of inflammasome genes, including NLRP3, ASC, and thereby caspase-1 and IL-1β.^22^ In addition, the pro-inflammatory form of cells, called pyroptosis, was also strongly inhibited by melatonin.^22^ (Fig. 1). Overall, melatonin triggers polarization to the anti-inflammatory (M2) phenotype, as shown by the upregulation of M2-specific markers, such as Arg1 and CD206, and disfavors the pro-inflammatory (M1) phenotype, as indicated by the downregulation of iNOS.^14^

In humans, infants with NE have altered immune circadian responses following ex vivo LPS stimulation, which has a potential for modulation.^23^ The circadian rhythm gene BMAL1 was significantly decreased with LPS stimulation in NE. Melatonin alters immune function ex vivo in infants and children. Melatonin ex vivo reduced neutrophil activation (CD11b) and Lipopolysaccharide recognition (LPS:Toll-like receptor-4 expression) in response to LPS in infants with NE compared to controls. In older school age children LPS-induced increases in systemic cytokines were abrogated (TNF-α, IL-8, IL-18, Epo and IL-1ra) by melatonin with more marked effects in the children with Down syndrome (DS).^24^ In the latter group there was a predominant pro-inflammatory immunophenotype with similarities to that of babies with NE. NLRP3 inflammasome and mRNA for Il1B were also significantly decreased by melatonin. In neonates with NE both NLRP3 and IL-1β gene expression were up-regulated in the presence of LPS and NLRP3 gene expression remained persistently increased in these children at school age compared to controls. Increased inflammasome activation in the first day of life in NE persists in childhood, and may increase the window for therapeutic intervention^25^ and be modified by melatonin as in animal models and also children with DS.^26^

This pilot study by Calero et al.^3^ in babies by with NE gives some suggestion that intravenous melatonin added to HT may effectively reduce pro-inflammatory levels, leading to improved long-term neurodevelopmental outcomes. Further research with larger sample sizes, with PK data is needed to confirm these findings. This research adds to the growing body of evidence supporting the potential of melatonin as a safe and effective adjunctive and single therapy for newborns with NE. The results, while preliminary, offer encouraging insights into the potential for melatonin to improve long-term neurological outcomes for these vulnerable infants.
